# Reporter Coxsackievirus A5 Expressing iLOV Fluorescent Protein or Luciferase Used for Rapid Neutralizing Assay in Cells and Living Imaging in Mice

**DOI:** 10.3390/v15091868

**Published:** 2023-09-02

**Authors:** Wei-Ping Jin, Chen Wang, Jie Wu, Jing Guo, Sheng-Li Meng, Ze-Jun Wang, Dai-Guan Yu, Shuo Shen

**Affiliations:** Wuhan Institute of Biological Products Co., Ltd., Wuhan 430207, China; jinweiping990@126.com (W.-P.J.); 18627716903@163.com (C.W.); wujie20230805@126.com (J.W.); guojingwh2004@hotmail.com (J.G.); mengshengli@sinopharm.com (S.-L.M.); wangzejun@sinopharm.com (Z.-J.W.); daiguanyu98@hotmail.com (D.-G.Y.)

**Keywords:** enterovirus, iLOV, nano luciferase, reporter enteroviruses, rapid neutralizing assay, living imaging

## Abstract

Coxsackievirus A5 (CV-A5) is a re-emerging enterovirus that causes hand, foot, and mouth disease in children under five years of age. CV-A5-M14-611 is a mouse-adapted strain that can infect orally and lead to the death of 14-day-old mice. Here, recombinants based on CV-A5-M14-611 were constructed carrying three reporter genes in different lengths. Smaller fluorescent marker proteins, light, oxygen, voltage sensing (iLOV), and nano luciferase (Nluc) were proven to be able to express efficiently in vitro. However, the recombinant with the largest insertion of the red fluorescence protein gene (DsRed) was not rescued. The construction strategy of reporter viruses was to insert the foreign genes between the C-terminus of VP1 and the N-terminus of 2A genes and to add a 2A protease cleavage domain at both ends of the insertions. The iLOV-tagged or Nluc-tagged recombinants, CV-A5-iLOV or CV-A5-Nluc, exhibited a high capacity for viral replication, genetic stability in cells and pathogenicity in mice. They were used to establish a rapid, inexpensive and convenient neutralizing antibody assay and greatly facilitated virus neutralizing antibody titration. Living imaging was performed on mice with CV-A5-Nluc, which exhibited specific bioluminescence in virus-disseminated organs, while fluorescence induced by CV-A5-iLOV was weakly detected. The reporter-gene-tagged CV-A5 can be used to study the infection and mechanisms of CV-A5 pathogenicity in a mouse model. They can also be used to establish rapid and sensitive assays for detecting neutralizing antibodies.

## 1. Introduction

Coxsackievirus A5 (CV-A5) is one of the re-emerging pathogens of hand, foot, and mouth disease (HFMD), herpangina and acute gastroenteritis, mainly affecting infants and young children [1,2,3,4]. CV-A5 is transmitted by the fecal–oral route and has caused sporadic or local epidemics previously in many countries, such as Japan, Thailand, China, Korea and Australia. Recently, it has emerged as one of the main serotypes associated with HFMD [1,4,5,6,7,8].

Epidemiological studies have shown a high proportion of HFMD cases caused by CV-A5 in Hang Zhou, Taiwan and Singapore [6,9,10]. Co-circulation of multiple serotypes of enteroviruses could increase the chances of coinfection and facilitate the emergence of new recombinants [2]. Following the immunization of children under five years with the inactivated, monovalent enterovirus 71 vaccine during 2016–2017 in Xiangyang, a high proportion of HFMD cases were caused by CV-A5. A CV-A5 isolated in Xiangyang is a prevailing, newly emerging recombinant CV-A5 carrying P2 and P3 gene sequences from an anther-prevailing CV-A2 strain [11].

CV-A5 is a member of the *Enterovirus* genus within the *Picornaviridae* family with a single, positive sense RNA genome at a length of approximately 7400 bases. A single open reading frame (ORF) encodes a polyprotein containing 2191 amino acid residues. The ORF is flanked by the 5′ and 3′ untranslated region (UTR) with a poly (A) tail. The polyprotein is cleaved by viral proteases 2A and 3C (or 3CD) to structural protein P1 and non-structural proteins P2 and P3. The P1 is further cleaved to VP0, VP3 and VP1, while P2 and P3 are cleaved to non-structural proteins 2A, 2B, 2C, 3A, 3B, 3C and 3D and intermediates 2BC, 3AB and 3CD. Procapsid (empty particle (EP)) is assembled by 60 copies of protomers composed of VP0, VP3 and VP1. It has been proposed that five protomers assemble into pentamers, viral RNA is encapsidated and twelve pentamers form provirion. VP0 is cleaved to VP4 and VP2 in a viral-RNA-dependent, autocatalytic process and, finally, forms an infectious virion (full particle (FP)), at 30 nm in diameter. In the cell culture of CV-A5, EP, FP and ‘A’ particles (or dense particle (DP)), an uncoating intermediate has been isolated and characterized [12].

There are no effective vaccines, therapeutic antibodies or antiviral drugs that can prevent infection caused by CV-A5 and treat patients. Previous research has proven that a formaldehyde-inactivated, whole-virus vaccine of CV-A5 stimulated an excellent humoral immune response to protect suckling mice challenged by a mouse-adapted strain CV-A5-M14-611 at a lethal dose [12]. However, the protection mechanism is not fully understood. Also, the neutralizing antibody titers in animal or human serum are determined by a traditional microneutralization assay via 50% inhibition of the cytopathic effect (CPE) following the incubation of a challenge virus with antibody or antiserum for 7 days. The conventional method is time-consuming and impractical for high throughput assays. CPE is judged by personal experience and is not accurate, especially when some enterovirus strains do not cause obvious and typical CPE.

The fluorescent-labeled tracing technique could be employed to monitor virus propagation and dissemination in vitro and in vivo. By inserting a gene encoding a fluorescent protein, a recombinant virus will release fluorescent labels in infected cells so that virus propagation and dissemination in experimental animals can be visualized and traced. 

Many researchers have shown that poliovirus, enterovirus 71, coxsackievirus A16, coxsackievirus B3, coxsackievirus B5 and Rhinovirus A within the *Picornaviridae* family have been engineered to carry reporter genes expressing fluorescent proteins and different kinds of luciferases [13,14,15,16,17]. These reporter viruses have been used to facilitate assays of antiviral reagents and in vivo imaging of mice [14,16,17]. However, one of the major challenges is that recombinants with large tagged genes are difficult to rescue and stably passaged in cells because of the limitation of insertion capacity of enteroviruses with a small genome of approximately 7.4 kilobases. 

Recently, iLOV (light, oxygen or voltage sensing) and nano luciferase (Nluc) have been exploited to construct reporter viruses for establishing detection methods [16,18,19]. iLOV has been applied to rhinovirus A and tobacco mosaic virus [20,21]. iLOV with a monomer is much smaller than green fluorescent protein (GFP) or red fluorescent protein (DsRed) and other fluorescent proteins that are used popularly in many basic studies. Smaller iLOV provides a reduced genetic load to overcome viral packaging constraints and stability during passaging, such as recombinants of enteroviruses. 

In this study, three different foreign genes encoding DsRed, Nluc and iLOV with different lengths were inserted into CV-A5 between the VP1/2A junction with cleavage sites at both ends of insertion. The efficiency of recombinant rescue, growth ability, genetic stability and pathogenicity of tagged recombinants were compared. CV-A5-Nluc and CV-A5-iLOV, but not CV-A5-DsRed, were successfully rescued and stably passaged in cells maintaining the biological characteristics of their parental strain. The results demonstrate that the size and locations of insertion are important for obtaining enterovirus recombinants with a reporter gene. The CV-A5-iLOV can be used in an inexpensive and convenient neutralizing antibody assay. The CV-A5-Nluc also can be used to improve neutralizing antibody assay and living tracing of virus dissemination in infected mice. 

## 2. Materials and Methods

### 2.1. Ethics Statement

Animal experiments were carried out in accordance with the guidelines for the standardization administration of China [22]. Experimental protocols were approved by the institutional animal care and use committee of the Wuhan Institute of Biological Products.

### 2.2. Cell Culture, Viruses and Antisera

Human rhabdomyosarcoma cells (RD) were maintained in Eagle’s minimal essential medium (MEM) (Nissui, Tokyo, Japan) supplemented with 10% newborn bovine serum (FBS) and were incubated at 37 °C in 5% CO_2_. The parental strain CV-A5-3487/XY/CHN/2017 (GenBank accession number MN663160) and the rabbit polyclonal antibody against CV-A5 FP were prepared as described previously [12]. The parental strain was mouse-adapted and named CV-A5-M14 (GenBank accession number MW079817). Plaque purification was performed and a plaque-purified, virulent clone was named CV-A5-M14-611 (abbreviated CV-A5-611 or 611). The murine serum against CV-A5 was also produced as described previously [12].

### 2.3. Viral RNA Extraction, cDNA Preparation and Construction of Recombinant Plasmids

The DsRed (717 bases), iLOV(330 bases) and Nluc (513 bases) with homological arms of the CV-A5 sequence were synthesized by Genscript Biotech. cDNA of CV-A5-611 was synthesized by primescript II 1st strand cDNA synthesis kit (Takara, Gunma, Japan) with oligo (dT) primers. The amplified cDNA template was used for constructing the full-length rCV-A5-611, CV-A5-611-DsRed, CV-A5-611-iLOV and CV-A5-611-Nluc cDNA clone. The recombinant pBR322-CV-A5-611 was constructed by joining the linearized plasmid and two PCR fragments covering the whole genome at a molar ratio of 1:2:2. The cloning strategy is illustrated in the Results section.

The plasmid pBR322-CV-A5-DsRed, pBR322-CV-A5-iLOV and pBR322-CV-A5-Nluc were constructed as described in the Results section. Two fragments 5′UTR-VP1 and 2A-3′UTR covering the complete genome were amplified from the reverse transcribed first strand cDNA with KOD DNA polymerase (TOYOBO, Tokyo, Japan). The linearized plasmid was obtained by digestion of the plasmid with MluI. Reporter genes DsRed, iLOV or Nluc were obtained through synthesis of the genes and amplification by PCR. The linearized pBR322, 5′UTR-VP1, inserted exogenous genes and 2A-3′UTR carrying 20 bp homological arms were joined for the construction of pBR322-CV-A5-DsRed, pBR322-CV-A5-iLOV and pBR322-CV-A5-Nluc. The four fragments were ligated at a molar ratio of 2:2:2:1 using an In-fusion HD Cloning kit (Takara, Gunma, Japan) and were chemically transformed into stellar competent Cells of *E. coli* (Takara, Gunma, Japan). All primers used for PCRs are listed in Table 1.

### 2.4. In Vitro RNA Transcription, Transfection and Virus Rescue

The recombinant plasmids were linearized with MluI (NEB, Ipswich, MA, USA) and the MluI restriction site was introduced into pBR322 downstream of the Poly (A) tail, as described previously [23]. The viral RNAs were transcribed by using the T7 high-yield RNA transcription kit (Promega, Fitchburg, WI, USA). The viral RNAs were transfected in 70% confluent RD cells with lipofectamine 3000 reagents (Thermo Fisher, Waltham, MA, USA) in 6-well plates and incubated at 37 °C in a 5% CO_2_ incubator (Thermo Fisher, Waltham, MA, USA). The supernatant containing rescued recombinant viruses named P0 was harvested.

### 2.5. Western Bolt Analysis

The supernatants of virus-infected RD cells were harvested and mixed with 4 × protein loading buffer. Proteins were separated by 1% SDS/4–20% gradient PAGE and were transferred onto 0.45 µm nitrocellulose membranes. The membranes were blocked for 30 min at 37 °C in PBS containing 1% (*W*/*V*) bovine serum albumin and 0.05% (*V*/*V*) Tween 20 (PBST-BSA). The membranes were incubated with rabbit CV-A5-FP antiserum at a dilution of 1:10,000 at 25 °C for 1 h. A horseradish peroxidase (HRP)-conjugated goat anti-rabbit secondary antibody (BSD, Wuhan, China) was added at a dilution of 1:10,000 in PBST-BSA and incubated for 45 min. The membranes were washed three times with PBST-BSA after each incubation step. The protein bands were detected with an ECL detection system (Gene, San Francisco, CA, USA). The membranes were stripped, blocked and incubated with an anti-actin antibody as loading control.

### 2.6. Plaque Assay

Confluent monolayer RD cells in 6-well plates were infected with ten-fold serial dilutions of virus stocks followed by 2 h incubation and overlaid with 2 mL per well of a mixture of 2% of low melting point agarose and 2 × MEM (*V*/*V* = 1:1). The plates were incubated for 72 h at 37 °C. The cells were fixed with 4% formaldehyde for 30 min. Low melting point agarose was removed and cells were stained by methylene blue for 30 min. The 6-well plates were rinsed with clean water and the images were photographed.

### 2.7. Growth Curves

Growth kinetics of the passage 2 (P2) of r611-iLOV and r611-Nluc were compared with that of the P2 r611. Briefly, 300 µL of the three viruses were inoculated into cells in 24 well plates (1 × 10^5^ cells/well) at a MOI of 10 and incubated at 37 °C. After absorption for 2 h, the supernatants were discarded and replaced with 2 mL per well MEM for incubation at 37 °C. At 4 h, 8 h, 12 h, 24 h, 28 h, 32 h, 36 h, 40 h, 44 h, 48 h, 52 h and 56 h post-infection, one of 12-well plates was removed. The virus cultures were subjected to three freeze-and-thaw cycles followed by centrifugation to remove cell debris for virus titration in triplicates. Virus titers in all supernatants were measured by 50% of cell culture infective dose (CCID_50_) assay and were calculated by the Spearman–Kärber equation.

### 2.8. Virulence of r611, r611-iLOV and r611-Nluc in Newborn Kunming Mice

To study whether the exogenous genes inserted in the viral genome affect virulence in mice, r611, r611-iLOV and r611-Nluc were inoculated into 14-day-old newborn Kunming mice via the intraperitoneal (i.p.) route at a dose of 1 × 10^7^ CCID_50_/mice. The control group was uninfected mice with equal volume MEM by i.p. route. The mice in each group were observed to monitor survival, body weight changes and clinical scores for two weeks. The criteria of clinical scores of infected suckling Kunming mice are the following: 0, healthy; 1, fatigue or sleepiness; 2, weight loss and hunched back; 3, hindlimb weakness or jitter; 4, hindlimb; 5, dying or death, as described previously [12].

### 2.9. Neutralization Assay

The basic and universal procedure for conventional neutralization assay and two improved neutralization assays were used. Viruses of r611, r611-iLOV or r611-Nluc were diluted in MEM (Nissui, Tokyo, Japan) so that 50 µL of the virus suspension contained 100 CCID50. An Equal volume (50 µL) of the 2-fold serially diluted antiserum was added to virus suspensions (2 duplicates for each dilution) in 96-well plates. After incubation at 37 °C for 2 h, 1 × 10^5^ RD cells in 100 µL of MEM with 10% FBS were added to the well. Positive and negative controls were included and virus back-titration was performed. The virus titers were in the range of 32 to 320 CCID_50_/50 µL. All neutralizing titers based on r611, r611-iLOV or r611-Nluc were calculated using the Reed–Muench method and expressed as the reciprocal of the highest serum dilution at which CPE or fluorescence or bioluminescence in 50% of the wells was completely inhibited. A neutralization titer of ≥8 would be regarded as positive.

For a conventional neutralizing assay to calculate serum titer, the CPE of cells in 96-well plates incubated with r611 and antiserum was checked under the microscope. iLOV neutralization method was developed to measure neutralizing antibody titers against r611-iLOV based on checking the fluorescence in the wells. After 48 h incubation of r611-iLOV and antiserum mixtures, supernatants were discarded and green fluorescence was detected by ELISPOT analyzers (Immunospot). Nano-Glo neutralization assay was developed to measure neutralizing antibody titers against r611-Nluc, based on a readout of the bioluminescence. After 48 h incubation of r611-Nluc and antiserum mixtures, the supernatant was discarded. Nano-Glo substrate solution was added following the manufacturer’s instructions (Promega, Fitchburg, WI, USA). Nano-Glo reagent was diluted at 1:20 in a diluted solution. Samples were transferred to a new white opaque 96-well plate (Corning, New York, NY, USA) and detected by multimode plate readers (PerkinElmer, Waltham, MA, USA).

### 2.10. Living Imaging of Mice via Fluorescence and Bioluminescence by Two Reporter-Tagged Viruses

Seven-day-old Kunming mice were infected by r611-iLOV or r611-Nluc via the oral route. All fluorescence or bioluminescence data were collected using an IVIS Lumina XRMS system (PerkinElmer, USA). All mice were anesthetized via isoflurane inhalation (5% isoflurane, oxygen flow rate of 1.5 L/min) prior to and during bioluminescence imaging (BLI) using the gas anesthesia system (Matrix, Newton, MA, USA). The nano-Glo substrate (Promega, Fitchburg, WI, USA) was diluted 1:20 in PBS. Each mouse in the groups was i.p. injected with 100 μL of the mixture 5–8 min before imaging. The mice were imaged in both dorsal and ventral views at days 1, 3 and 6 post infection. Images were acquired and analyzed with living image in vivo software (PerkinElmer, USA). Photon flux was measured as luminescent radiance (p/sec/cm^2^/sr).

### 2.11. Statistical Analysis

All data were analyzed with GraphPad Prism 8 software. Statistical significance was assessed using a one-way ANOVA multiple comparison test.

## 3. Results

### 3.1. Construction and Rescue of the Recombinant CV-A5-611 Mouse-Adapted Strain

**Figure 1:**
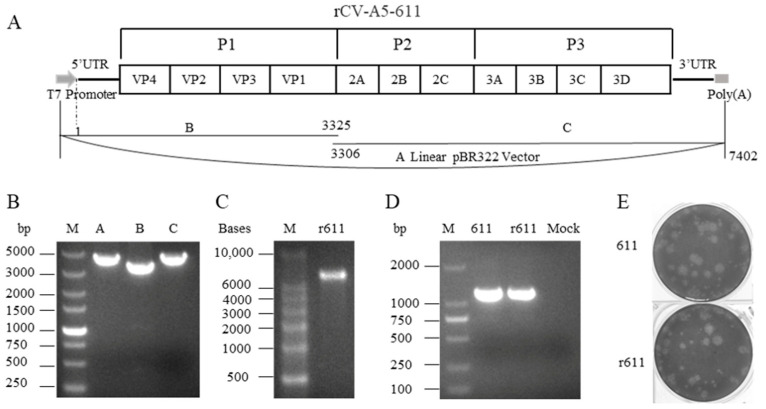
Construction and rescue of recombinant mouse-adapted CV-A5-611. (**A**) Genome organization and construction strategy of the infectious cDNA clone of rCV-A5-611 are shown. Fragment A represents linearized pBR322 vector. Two PCR fragments B (T7 promotor + nucleotides 1–3325) and C (nucleotides 3306–7402 + polyA_25_) represented by black lines were amplified from the first strand cDNA by PCR with specific primer pairs. (**B**) The amplified fragments A, B and linearized plasmid were detected by agarose gel electrophoresis. (**C**) Detection of the in vitro transcribed recombinant virus RNA from an insert-positive plasmid after the denature at 65 °C, 5 min. (**D**) Viral RNAs were extracted from RD cells infected with rescued rCV-A5-611 and parental CV-A5-611 and PCR fragments amplified using specific primers of virus genes were detected in gel electrophoresis. Mock represents RT-PCR using RNA extracted from uninfected cells. (**E**) Virus plaque assay of rescued rCV-A5-611 and parental virus CV-A5-611 in RD cells on day 3 post infection.

The CV-A5-611 is a virulent strain plaque-purified from a mouse-adapted CV-A5-M14 that is lethal to two-week-old Kunming mice, as described previously [12]. To construct the infectious cDNA, viral RNA was extracted from the CV-A5-611 and reverse-transcripted to obtain the first strand cDNA as templates for PCR amplification. The construction schematic is shown in Figure 1A. Two viral gene fragments B and C were amplified from the cDNA and linear fragment pBR322 (fragment A) was amplified from the circular plasmid by PCR (Figure 1B). The three fragments were joined by the in-fusion enzyme to obtain a recombinant CV-A5 plasmid. A T7 promoter sequence was engineered upstream of the 5′-UTR of the CV-A5 genome as previously described [23]. Mlu I restriction enzyme site was inserted to obtain a linearized plasmid. The linearized plasmid of the recombinant plasmid was used as a template for in vitro transcription. Analysis of the full-length viral RNA transcribed in vitro by gel electrophoresis showed that the size was correct as expected (Figure 1C). After transfection of transcribed viral RNA in RD cells, recombinant rCV-A5-611 was rescued. The PCR fragments of parental CV-A5-611 and rescued rCV-A5-611 were amplified (Figure 1D) and sequenced to be correct. There is no significant difference in plaque size and morphology between parental CV-A5-611 and recombinant rCV-A5-611 (Figure 1E). All results showed the infectious cDNA of CV-A5 was successfully constructed.

### 3.2. Construction and Rescue of the Recombinant CV-A5-611 Tagged with Reporter Genes

**Figure 2:**
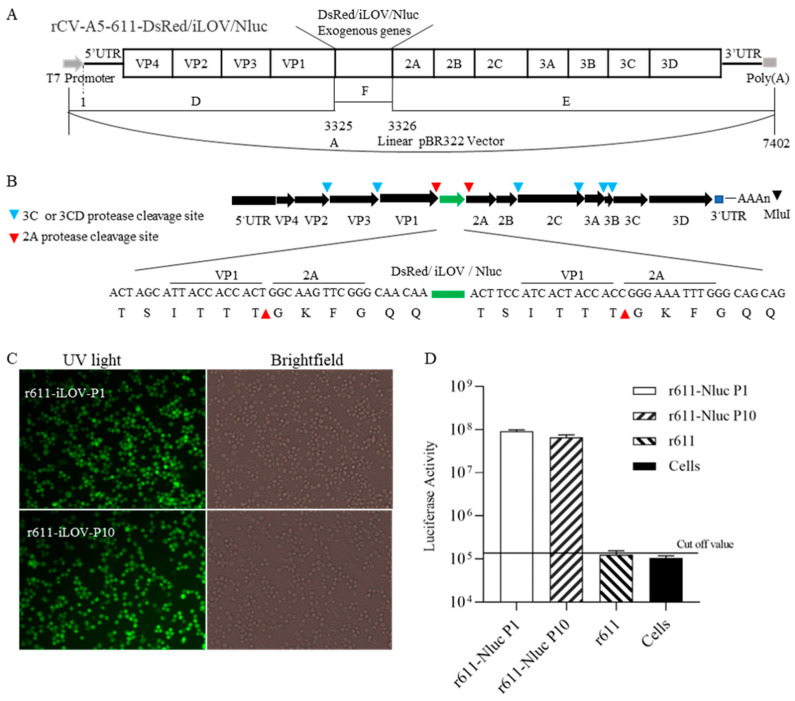
Schematic representation for construction and rescue of the CV-A5 reporter viruses. (**A**) Construction strategy of the CV-A5-611-DsRed, CV-A5-611-iLOV and CV-A5-611-Nluc reporter viruses. Fragment A represents linear pBR322 vector. Fragments D (T7 promotor + nucleotides 1–3325) and E (nucleotides 3326–7402+ polyA_25_) indicated by lines were amplified from the first strand cDNA by specific primer pairs. Fragment F was amplified by PCR that represents DsRed, iLOV or Nluc gene with homological arms, respectively, for linking to fragments D and E. (**B**) DsRed or iLOV or Nluc gene was inserted between cleavage site of VP1/2A of 2A protease. The TSITTTGKFGQQ represents amino acid residues up- and downstream of 2A protease cleavage site TG that was introduced into pBR322-CV-A5-611 for expression of the exogenous reporter genes. (**C**) The r611-iLOV passages P1 and P10 were detected. (**D**) Bioluminescence induced by r611-Nluc P1 and P10 was quantified after adding nano-Glo substrate solution. The background bioluminescence was defined by the cut-off value induced in the uninfected cell.

Based on rCV-A5-611, the construction schematic of recombinant reporter viruses is shown in Figure 2A. iLOV or DsRed or Nluc gene was inserted into VP1 and 2A junction with the upstream and downstream sequences encoding “TSITTT↓GKFGQQ” containing 2A protease cleavage site TG. Up to 6 residue addition on both sides of TG was designed for efficient release of reporter from the polyprotein (Figure 2B). For example, when the CV-A5-iLOV viral genome was translated to a polyprotein, 2A protease recognized the cleavage site “TSITTT↓GKFGQQ”, leading to the release of the exogenous protein, and virus assembly was not affected. To rescue infectious recombinants, 5 µg in vitro transcripts of viral RNA of 611-DsRed, 611-iLOV and 611-Nluc were transfected into, respectively, RD cells as described in materials and methods. Obvious CPEs caused by r611-DsRed, r611-iLOV and r611-Nluc were observed (Figure 2C). Green fluorescence caused by CV-A5-iLOV after infection with passages 1 and 10 (P1 and P10) was observed. However, red fluorescence DsRed caused by rCV-A5-DsRed was transiently observed in P0 and was absent in cells infected with passage P1 (Figure 2C), indicating that CV-A5 tagged with DsRed was not stable. Bioluminescence after adding substrates was continuously detected in cells infected with r611-Nluc passages P1 and P10 (Figure 2D). The results showed that the construction strategy could rescue CV-A5-r611-iLOV and CV-A5-r611-Nluc successfully, but failed to rescue CV-A5-r611-DsRed in RD cells. It was noticed that six extra residues in both ends of iLOV and Nluc, designed for efficient release of inserts from polyprotein, did not affect the signal strength.

### 3.3. Genetic Stability and Particle Assembly of Reporter Viruses in Cell Culture

**Figure 3:**
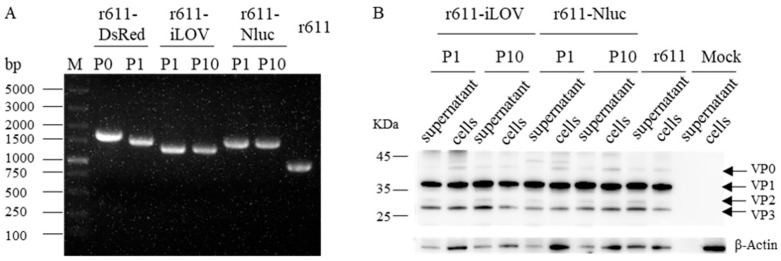
Genetic stability of tagged CV-A5 recombinants. (**A**) RNAs were extracted from cells transfected with viral RNA (P0) and infected with passage P1 of r611-DsRed, infected with passages 1/10 of r611-iLOV and r611-Nluc and parental r611. PCR fragments were amplified from reversely transcribed RNAs using specific primers to cover the exogenous gene and partial CV-A5 sequences and analyzed on agarose gel. (**B**) Viral structural proteins of the rescued r611-iLOV P1, P10, r611-Nluc P1, P10 and r611 recombinants in both infected cells and cultured supernatants were analyzed by Western blotting using anti-CV-A5 antiserum. The supernatants were detected directly while the cell lysates were diluted 10 fold before loading. VP0, VP1, VP2 and VP3 are indicated with arrows. Molecular weight markers are indicated with lines (in base pair and kilodalton). The loading control was included using an anti-β-Actin antibody. Parental virus r611-infected and mock-infected RD cells were used as controls.

To further investigate the genetic stability of exogenous reporter genes tagged in recombinants in the process of sequentially passaging in RD cells, PCR fragments were amplified and viral protein expression was analyzed in transfected cells and infected cells with recombinants of different passages. As shown in Figure 3A, the intact exogenous genes iLOV and Nluc were stably retained in r611-iLOV and r611-Nluc genomes over ten passaging. However, the size of the PCR fragment of r611-DsRed P1 was smaller than that of P0. Western blotting showed that the structural proteins VP1, VP2 and VP3 of r611-iLOV and r611-Nluc, like r611, were detected using rabbit anti-CV-A5-FP serum in both infected cells and cultured supernatants after passaging 10 times (Figure 3B). No difference in the efficiency of P1 cleavage of tagged viruses was observed, compared with untagged r611. Exogenous genes of iLOV and Nluc inserted into the genome did not affect the genetic stability, polyprotein cleavage and particle assembly in recombinant passage 10. Sanger sequencing analysis of viral genomes with the iLOV and Nluc gene insertions revealed that no mutations and small insertion and deletion occurred. However, the DsRed gene sequence was partly deleted and the ORF of CV-A5-r611-DsRed was broken. The results were consistent with the results that green fluorescence from infected RD cells was not weakened between passages 1 and 10 of r611-iLOV (Figure 2C). Also, the high levels of bioluminescence of r611-Nluc passages P1 and P10 in infected cells were detected and the nano-luciferase protein is stably and strongly expressed (Figure 2D). 

### 3.4. Propagation in Cells and Virulence of r611-Nluc and r611-iLOV in Mice

**Figure 4:**
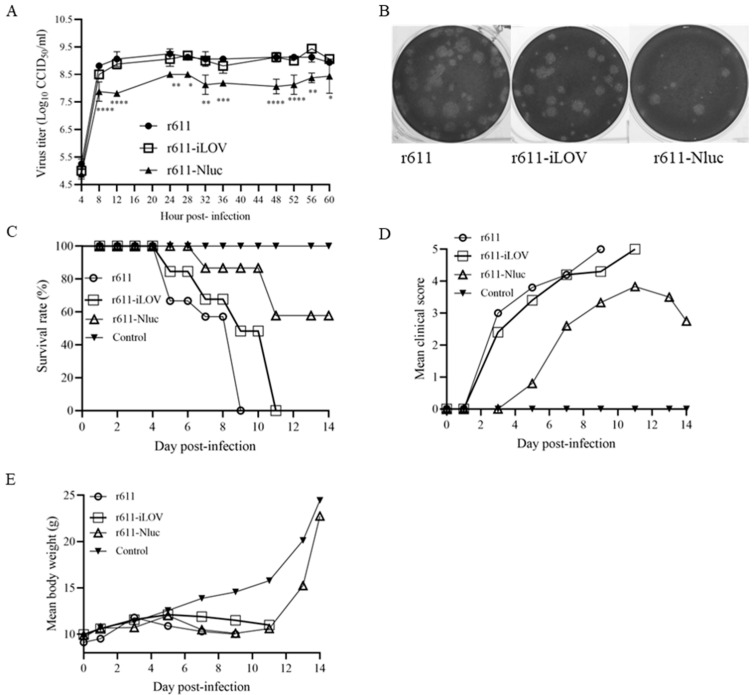
Growth ability and virulence of r611-iLOV and r611-Nluc. (**A**) Comparison of the growth kinetics of recombinants was performed on RD cells infected with r611-Nluc, r611-iLOV and r611 at the same M.O.I. (MOI = 10) *, **, ***, and **** indicate *p* < 0.05, *p* < 0.01, *p* < 0.001 and *p* < 0.0001 (using one-way ANOVA), respectively. (**B**) Plaque formation of r611, r611-iLOV and r611-Nluc was observed on RD cells on day 3 post infection. (**C**) The 14-day-old suckling Kunming mice were infected with r611, r611-iLOV and r611-Nluc and their survival rates were calculated 14 days post infection. (**D**,**E**) Mean clinical scores and body weight changes of the recombinants were recorded during the 14-day observation period post infection.

Further, to investigate the growth ability and virulence of iLOV- and Nluc-tagged recombinants, growth curves in RD cells and pathogenicity in mice of the recombinants were performed. The insertion of the iLOV gene in the genome of r611-iLOV did not affect growth ability as the virus titers were the same compared with rCV-A5-611 at different time points (Figure 4A). However, the growth ability of r611-Nluc decreased as the virus titers of the r611-Nluc were significantly lower than those of rCV-A5-611 (Figure 4A). The virus titers of r611-Nluc decreased by approximately 10 fold compared to those of rCV-A5-611. The results demonstrated again that r611-iLOV and r611-Nluc were genetically stable during the replication in RD cells. r611-iLOV and r611-Nluc showed no differences in plaque size compared with CV-A5-r611 in RD cells (Figure 4B).

The 14-day-old Kunming mice were infected with r611-iLOV, r611-Nluc and r611 at the same challenge dose (1 × 10^7^ CCID_50_/mouse) for comparison of virulence. The survival rate of the control group with MEM is 100% without clinical symptoms and weight loss. The survival rates of r611-iLOV were higher than those of r611 from day 5 to day 10 post-infection. All mice inoculated with r611-iLOV died on day 11, while all mice inoculated with r611 died on day 9 (Figure 4C). Therefore, the r611-iLOV with insertion of the exogenous gene could be still used as a challenging virus, though the insertion affects the virulence in newborn Kunming mice slightly. However, the survival rates of r611-Nluc were much higher than those of r611-iLOV and r611 at different time points (Figure 4C), suggesting that a higher infection dose should be used for 100% of mortality. The mean clinical scores of r611-iLOV and r611 were the same, but both were higher than those of r611-Nluc and caused milder symptoms compared to r611 (Figure 4D). There were no differences in body weight changes among the three viruses (Figure 4E). Inserting the Nluc gene affected the virulence in Kunming mice, probably due to a reduction in the growth rate. r611-Nluc can be 100% lethal to 12-day-old Kunming mice at the dose of 1 × 10^7^ CCID_50_/mouse , suggesting that the ages of mice were related to virulence.

### 3.5. Neutralization Assay Using r611, r611-iLOV and r611-Nluc of CV-A5

**Figure 5:**
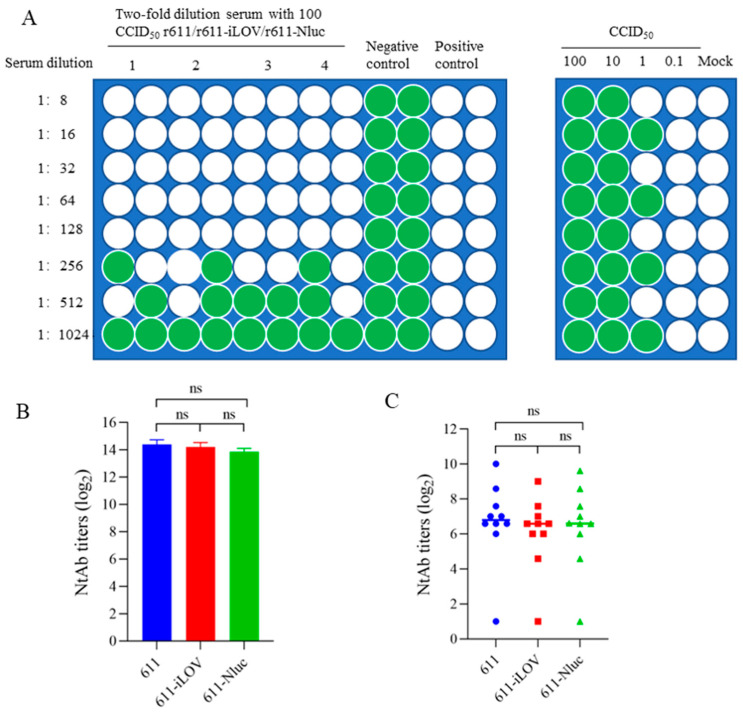
Microneutralization assay based on r611-iLOV and r611-Nluc for rapid analysis of neutralizing antibody levels in serum samples. (**A**) Schematic representation of a method designed for performing antibody neutralization assay in a format of 96-well plates (left panel). For example, serum neutralizing titers were determined to be 384 by 4 times repeated experiments. Green and white wells represent fluorescence, caused by infection of r611-iLOV, with CPE and fluorescence-free without CPE, respectively. All viruses were back-titrated to make sure that viruses of 30-320 CCID_50_ were used (right panel). Mock represents uninfected normal cell controls. (**B**) The rabbit serum titers were detected by the r611-iLOV, r611-Nluc and r611 using iLOV fluorescence, luciferase and conventional neutralizing assays, respectively, for comparison. (**C**) The mouse serum titers were determined by the r611-iLOV, r611-Nluc and r611. Each symbol represented a mouse. The titers of rabbit and mouse antisera based on r611 (assayed by conventional method) were calculated on day 7, while those based on r611-iLOV and r611-Nluc on day 2 post-neutralizing. One-way ANOVA was used for statistical significance analysis. ns, not significant, *p* > 0.05.

The r611-iLOV and r611-Nluc were used to titrate neutralizing antibody titers of antisera and r611 was used for comparison. The serum derived from Japanese long-eared white rabbits immunized with purified CV-A5 was obtained as previously described [12]. The titer of rabbit serum was determined by r611-iLOV and r611-Nluc (Figure 5A). For comparison, r611 was used for titration of the same antisera by conventional method. Titers are averages of four repeated experiments. The neutralizing titers of the serum based on two reporter viruses on day 2 are not significantly different compared with r611 on day 7 by conventional titration assay (Figure 5B).

Antisera from ten mice was obtained from mice immunized with a formalin-inactivated CV-A5 vaccine as previously described [12]. The seroconversion rate of the ten mice measured by r611-iLOV and r611-Nluc on day 2 was 90%, equal to that by r611 on day 7 (Figure 5C). The titers of nine positive sera were distributed equally from 32 to 1024 using the three viruses. The titers of mouse antisera were determined by three neutralization viruses by the same format and count method.

Taken together, the titers based on r611-iLOV and r611-Nluc were slightly lower than those based on r611 due to the sensitivity of the methods. The result showed that neutralizing titers based on r611-iLOV on day 2 had no significant difference compared with those based on r611 on day 7 (Figure 5B,C). Neutralizing assay using r611-iLOV has an advantage over using r611-Nluc where an expensive substrate is needed.

### 3.6. Living Imaging of Mice Infected with r611-iLOV and r611-Nluc

**Figure 6:**
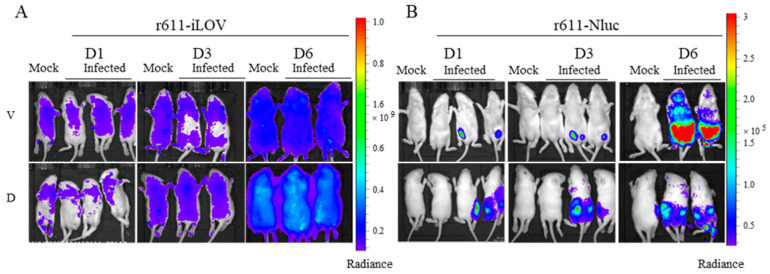
In vivo imaging of CV-A5-r611-iLOV and CV-A5-r611-Nluc infected Kunming mice. (**A**) Fluorescence measurement of Kunming mouse infected with r611-iLOV at days 1, 3 and 6 post infection. Mock represents the uninfected mouse inoculated with equal volume of PBS. Color scale, Min = 9.50 × 10^7^, Max = 1.00 × 10^9^. (**B**) Bioluminescence measurement of Kunming mouse infected with r611-Nluc at days 1, 3 and 6 post infection using furimazine substrate. Color scale, Min = 3.00 × 10^4^, Max = 3.00 × 10^5^. Mock represents the uninfected mouse inoculated with equal volume of PBS and substrate. Radiance, p/sec/cm^2^/sr. D, dorsal; V, ventral.

To test whether two reporter viruses could be used for living imaging in the mouse model, 7-day-old suckling mice were orally infected with r611-iLOV and r611-Nluc at the same dose of 1 × 10^6^ CCID_50_ per mouse. The results showed that the iLOV protein was not suitable for in vivo imaging because of the lack or weak fluorescence in mice infected with r611-iLOV (Figure 6A). Specific fluorescence in the organs of mice was not observed compared with mock-infected mice and virus dissemination could not be monitored, though severe clinical symptoms showed in infected mice at days 3 and 6 post infection. The in vivo imaging research used by r611-iLOV should be optimized through further modification of iLOV and improvement of design. In contrast, the mice infected with r611-Nluc showed specific bioluminescence and virus dissemination was observed from day 1 to day 6 post infection, compared to the mock-infected mice (Figure 6B). No signal was detected when mice were inoculated with an equal volume of PBS, suggesting a very low background during the in vivo imaging. The hindlimb paralysis symptom was caused by the tagged coxsackievirus A5. For the viral spreading, one dying mouse infected by r611-Nluc showed severe hindlimb paralysis and strong specific bioluminescence detected on day 6 (Figure 6B). The results demonstrate that the r611-Nluc can be used for both in vivo and in vitro studies on replication and pathogenesis.

## 4. Discussion

In this study, CV-A5 recombinants were constructed by inserting the iLOV and Nluc genes between the C-terminus of VP1 and the N-terminus of 2A, harboring sequences encoding extra residues at the cleavage site TG (TSITTTGKFGQQ) of 2A protease at both sides of insertion. An iLOV reporter CV-A10 was also established in our laboratory using the same strategy as CV-A5 and used for rapid screening of mouse hybridomas secreting neutralizing monoclonal antibodies. It is worth noting that the reporter-tagged CV-A10 was also stable and viable after being passaged ten times at least in RD cells. This strategy could be used on other enteroviruses in the *Enterovirus* genus for study on mechanisms of virus replication and pathogenesis or rapidly high throughput neutralizing antibody detection. It is worth noting that the wobble base mutation of the 2A cleavage domain, encoding TSITTTGKFGQQ, was introduced to construct the recombinant viruses by the highly efficient amplification of the PCR product. The 2A cleavage domain translated by wobble base mutation can still be recognized and cleaved successfully, indicating that the use of wobble base mutation did not interfere with normal translation and cleavage by the 2A protease.

We also tried to construct the CV-A5 recombinant tagged with reporter genes that were inserted in a short 2A cleavage domain around the TG site (ITTT↓G) in front of the start codon of the VP4 gene by the same strategy, as described previously [14,17]. Stably recombinants were not obtained as the exogenous insertions eGFP or DsRed were deleted during the passaging. Also, exogenous genes inserted downstream of the 3CD failed to rescue stable recombinants. Presumedly, the sizes of the eGFP or DsRed insertions exceed the limitation of the packaging capacity of CV-A5. It is also possible that the locations of insertion influence the secondary structures of CV-A5 which is critical to the encapsidation of the viral genome [24]. The iLOV (330 bases) sequence encoding a monomer domain of phototropin is smaller than those of eGFP (717 bases), DsRed (678 bases) and Nluc (513 bases). 

Previous research has shown that influenza A virus, foot and mouth disease viruses, Senecavirus A, West Nile virus and dengue virus have been engineered to carry a reporter gene for high throughput monoclonal antibody and antiviral assays [25,26,27,28,29,30]. Tagged EV-A71 and CV-A16 of the *Enterovirus* A are constructed by the same strategy to insert the GFP, nano luciferase, or gaussia luciferase gene between the 5′-UTR and the N-terminus of VP4, with the addition of a 2A protease cleavage site (ITTLG) at C-terminus of the 5′-UTR [13,14,15,16,17]. CV-B3 was constructed as a stable expression vehicle for GFP released from the viral polyprotein by 2A^pro^ cleavage. CV-B3 with inserted exogenous genes is an essential tool for viral tracking and gene delivery to humans. Coxsackievirus B5 viruses with nano luciferase were constructed to study viral protein translation and viral replication with 3C protease self-cleavage site (ALFQG) [19]. Rhinovirus (RV) usually causes the common cold and asthma exacerbations that belong to the *Picornaviridae* family, genus *Enterovirus*. Previous work showed that RV1A-iLOV could be used in studies of pathogenesis. The result suggests that the strategy may be applicable to other enterovirus serotypes and other viruses belonging to the genus *Enterovirus*.

At present, the photosensitive protein iLOV has been successfully applied to Rhinovirus, Senecavirus A, JC virus, Getah virus, barley stripe mosaic virus, porcine astrovirus and Rose Rosette Virus to study their pathogenesis and molecular biology on the cellular level, and screening antiviral drugs [20,27,31,32,33,34,35]. The smaller size of the iLOV coding sequence is likely to be used extendedly to a range of fluorescently tagged plant and animal viruses, facilitating the study of the trafficking of viruses in vivo. 

Even if the fluorescent protein gene is successfully inserted into the genome of a small-size enterovirus, the RNA encoding a fluorescent protein would be deleted when the virus replicates. Meanwhile, the insertion of foreign genes makes the genome of the RNA virus unstable and not viable, affecting the replication and assembly. Compared with luciferase imaging, the prominent advantage of iLOV fluorescent protein imaging is that it does not require an injection of exogenous substrates for study in vivo and in vitro. Data in this study indicate that the maximum packaging capacity of enteroviruses is approximately 500 bases (Nluc) and that extra residues around the cleavage site of 2A^pro^ ensure the efficient and complete processing of viral polyprotein. Enteroviruses inserting exogenous genes have potential prospects in the field of oncolytic gene therapy [36]. 

In summary, infectious CV-A5 recombinants with reporter genes based on a mouse-adapted strain were generated using the smaller iLOV and Nluc genes. The strategy designed would be highly efficient to construct an infectious CV-A5 with tagged genes. The CV-A5-iLOV and CV-A5-Nluc reporter viruses have little, if any, effect on viral viability, fitness, genome stability, assembly and virulence following passaging in cells for at least ten passages. The neutralization assay based on CV-A5-iLOV and CV-A5-Nluc reporter developed in this study should be useful for a novel, high throughput and rapid screening assay for mouse or human neutralizing monoclonal antibodies. A mouse-adapted, orally administrated, virulent CV-A5-Nluc would be applied to live imaging in infected mice. The reporter viruses of CV-A5-iLOV and CV-A5-Nluc based on a virulent strain also would be a useful tool for studying CV-A5 replication and pathogenesis, evaluating the efficacy of different type vaccine candidates and antiviral drugs.

## 5. Patents

There is a patent resulting from the work reported in this manuscript (Chinese patents approved number: CN202211012539.X).

## Figures and Tables

**Table 1:** Primers used in this study.

Primers	Fragment	Sequence (5′–3′) and Nucleotide Positions of CV-A5 Genome
pBR322-F	A	**ACGCGTGGATCCTCTACGCC**GGACG
pBR322-R		**CCCTATAGTGAGTCGTATTA**ACTAGTAAGCTTATCGATGATAAGCTGTCAAACA
T7-5UTR-F	B	**TAATACGACTCACTATAGGG**TTAAAACAGCCTGTGGGTTGTACCCACCC, (T7 promotor + Nucleotides 1–29)
611-VP1-2A-R		**AGTGGTGGTAATGCTAGTCC**TATTAAATGAAGCGTCCACAATATT,(Nucleotides 3281–3325)
611-LOV-VP1-2A-F	C	**GGACTAGCATTACCACCACT**GGGAAATTTGGGCAGCAGTCAG, (Nucleotides 3306–3347)
pBR322-3UTR-R		**GGCGTAGAGGATCCACGCGT**TTTTTTTTTTTTTTTTTTTTTTTTGCTATTCTGGTTATA, (Plasmid 1–14+ MluI+ Poly A_25+_Nucleotides 7388–7402)
T7-5UTR-F	D	**TAATACGACTCACTATAGGG**TTAAAACAGCCTGTGGGTTGTACCCACCC, (T7 promotor + Nucleotides 1–29)
611iLOV-VP1-2A-R		**AGTGGTGGTAATGCTAGTCC**TATTAAATGAAGCGTCCACA, (Nucleotides 3286–3325)
611iLOV-LOV-VP1-2A-F	E	**GGGAAATTTGGGCAGCAGTC**AGGAGCTGTGTATGTGGGAA, (Nucleotides 3326–3365)
pBR322-3UTR-R		**GGCGTAGAGGATCCACGCGT**TTTTTTTTTTTTTTTTTTTTTTTTGCTATTCTGGTTATA, (Plasmid 1–14+ MluI+ Poly A_25+_Nucleotides 7388–7402)
611 DsRed-VP1-DsRed-F	F (for DsRed)	**GGACTAGCATTACCACCACT**GGCAAGTTCGGGCAACAAATGGCCTTCTCCGAGGACGTCATC, (Nucleotides 3305–3325 + 2A 3326–3343 + DsRed 1–24)
611 DsRed-VP1-DsRed-R		**GACTGCTGCCCAAATTTCCC**GGTGGTAGTGATGGAAGTGGCGCCGGTGGAGTGGCGGCCCTCGG, (Nucleotides 3326–3345+ VP1 3308–3325 + DsRed 692–717)
611iLOV-VP1-iLOV-F	F (for iLOV)	**GGACTAGCATTACCACCACT**GGCAAGTTCGGGCAACAAATCGAGAAGAACTTCGTGATCACC, (Nucleotides 3305–3325 + 2A 3326–3343 + iLOV 1–24)
611iLOV-VP1-iLOV-R		**GACTGCTGCCCAAATTTCCC**GGTGGTAGTGATGGAAGTCACGTGGTCGCTGCC, (Nucleotides 3326–3345+ VP1 3308–3325 + iLOV 316–330)
611Nluc-VP1-Nluc-F	F (for Nluc)	**GGACTAGCATTACCACCACT****GGCAAGTTCGGGCAACAA**ATGGTCTTCACACTCGAGGAT, (Nucleotides 3305–3325 + 2A 3326–3343 + Nluc 1–21)
611Nluc-VP1-Nluc-R		**GACTGCTGCCCAAATTTCCC**GGTGGTAGTGATGGAAGTCGCCAGAATGCGTTCGCACA, (Nucleotides 3326–3345+ VP1 3308–3325 + Nluc 494–513)

The bold fonts indicate homological arms for constructing recombinant viruses. The underlined fonts represent CV-A5 genome sequence. The double underlined fonts represent wobble base mutation of VP1 or 2A protease.

## Data Availability

No supporting data.
